# Numerical Simulation and Experimental Study of Deposition Behavior for Cold-Sprayed Nano-Structured HA/70wt.%Ti Composite Coating

**DOI:** 10.3390/nano15231807

**Published:** 2025-11-29

**Authors:** Xiao Chen, Chengdi Li, Shuangxia Zhu, Peiyun Ao, Yao Hu

**Affiliations:** Xinyu Key Laboratory of Materials Technology and Application for Intelligent Manufacturing, School of Mechanical and Electrical Engineering, XinYu University, Xinyu 338004, China; chenxiaoxyxy@126.com (X.C.); zhushuangxiaxyyx@163.com (S.Z.); aopeiyunxyxy@163.com (P.A.); yaohu1990_xyxy@163.com (Y.H.)

**Keywords:** cold spray, HA/Ti coating, numerical simulation, deposition behavior

## Abstract

This study employs numerical simulations and experiments to examine the cold spray deposition of nanostructured hydroxyapatite (Ca_10_(PO4)_6_(OH)_2_, HA)/70wt.%Ti composite particles under different processing conditions, based on the features of nanocomposites that strengthen interfacial adhesion and improve coating interfacial strength. Using ABAQUS/CAE combined with LS-PrePost 4.9-x64 software, the deposition behavior of the composite particles during deposition under various impact velocities was analyzed, along with the stress of the HA and Ti particles within the composite particle. The deposition behavior of both single and multiple composite particles under different gas temperatures was studied through cold spray experiments, and composite coatings were fabricated. The microstructure and phase composition were analyzed using scanning electron microscopy (SEM) and X-ray diffraction (XRD). The results showed that the numerical simulations were consistent with the experimental analyses. As the particle velocity or gas temperature increased, the degree of particle deformation upon deposition became more pronounced, accompanied by phenomena such as cracking or fragmentation and splashing rebound. At a gas temperature of 700 °C, both the bonding density of individual particles and the bonding effectiveness of multi-particle deposits were lower than those achieved at 500 °C. The coating prepared at a gas temperature of 500 °C exhibited a flatter surface, better overall bonding with the Ti interlayer, and higher internal density.

## 1. Introduction

In addition to demonstrating certain biological properties, bone repair materials must also exhibit excellent mechanical properties and interfacial bonding [1,2]. Hydroxyapatite (Ca_10_(PO4)_6_(OH)_2_, HA) is commonly employed as a biocoating material for the surface modification of titanium and its alloys due to its biological properties [3,4]. However, the application of pure HA materials in load-bearing areas of the human body is significantly limited due to poor mechanical properties, substantial physicochemical differences between the substrate and the body, and low interfacial bonding caused by issues such as insufficient crystallinity or phase transition/decomposition during the preparation process [5]. Research has demonstrated that in nanocomposites, the introduction of nanoparticles with an elevated specific surface area promotes interfacial strengthening between phases, while grain size reduction leads to corresponding enhancements in interfacial bonding strength [6]. In contrast to conventional micro-HA, nano-HA offers better mechanical performance in tissues and promotes stronger interfacial integration [6,7]. Additionally, due to the superior mechanical properties of Ti, the incorporation of nano-Ti particles into nano-HA can achieve reinforcement and toughening effects, while mitigating residual stress between the coating and substrate to enhance interfacial bonding strength [8].

The current the preparation methods for HA composite coatings primarily include magnetron sputtering [9,10], electrochemical deposition [11,12], laser cladding [13,14,15], and thermal spraying [3,4,16,17,18,19], among others. However, due to the challenges involved in controlling the composition and crystallinity of HA coatings or the presence of significant residual stresses, it is difficult to effectively improve the interfacial bonding strength of the coatings. As a result, the performance of HA composite coatings still struggles to meet the application requirements for bone repair materials. Thus, a novel and highly efficient technique for the fabrication of HA composite coatings must be developed to enable precise control over their microstructure, composition, and crystallinity.

Cold spraying is a coating deposition method based on the low-temperature solid-state plastic deformation of high-kinetic-energy particles upon impact with the substrate surface [20]. It has achieved applicable progress in the preparation of metal [20,21,22,23], alloy [24], and ceramic [25] coatings. Moreover, owing to its low-temperature deposition characteristics, cold spraying has emerged as a novel technology for fabricating coatings from thermally sensitive materials, such as WC-Co cermets [26,27,28,29], HA bioceramics [30,31,32], and nano-structured materials [26,29,30,32,33]. Compared with thermal spraying technology, cold spraying can transfer the original microstructure of the powder to the coating, which not only preserves the inherent structure of the sprayed material but also avoids issues such as decomposition, phase transformation, and grain growth in nanostructured materials caused by high-temperature [26]. Furthermore, the compressive stresses induced by cold spray deposition can significantly improve interlayer adhesion. By precisely controlling the deformation and bonding behavior of particles during deposition, it becomes feasible to regulate both coating porosity and interparticle interfacial bonding [27]. This offers a novel approach for fabricating HA composite coatings with diverse compositions and microstructures.

At present, numerous researchers are focusing primarily on experiments on the deposition behavior of cold-sprayed micronstructured HA/Ti (mHA/Ti) composite particles [34]. However, there is relatively limited research on the cold spray deposition behavior of nanostructured HA/Ti (nHA/Ti) composite particles, especially in the area of numerical simulations. Therefore, based on the characteristics of low-temperature deposition and particle impact compaction in cold spraying for enhancing interfacial bonding, as well as the features of nanocomposites that strengthen interfacial adhesion and improve coating interfacial strength through the addition of second-phase nanoparticles, the deposition behavior of nHA/Ti using both experimental and numerical simulation approaches was investigated. The main innovations and research objectives of this study are as follows: (1) dual-nano HA/Ti composite powder was successfully prepared using nano-HA and nano-Ti; (2) Numerical simulations of the deposition deformation of the dual-nano HA/Ti composite particles were conducted at different particle velocities to analyze the deformation mechanism and the stress evolution of both nano-HA and nano-Ti particles during deposition; (3) The bonding behavior during single- and multi-particle deposition of HA/Ti composites was examined using cold spray technology at different gas temperatures, thereby providing experimental validation for the numerical simulation results; (4) nanostructured HA/Ti composite coatings were successfully fabricated via cold spraying under varying gas temperatures, and the underlying coating bonding mechanism was elucidated.

## 2. Materials and Methods

**Figure 1:**
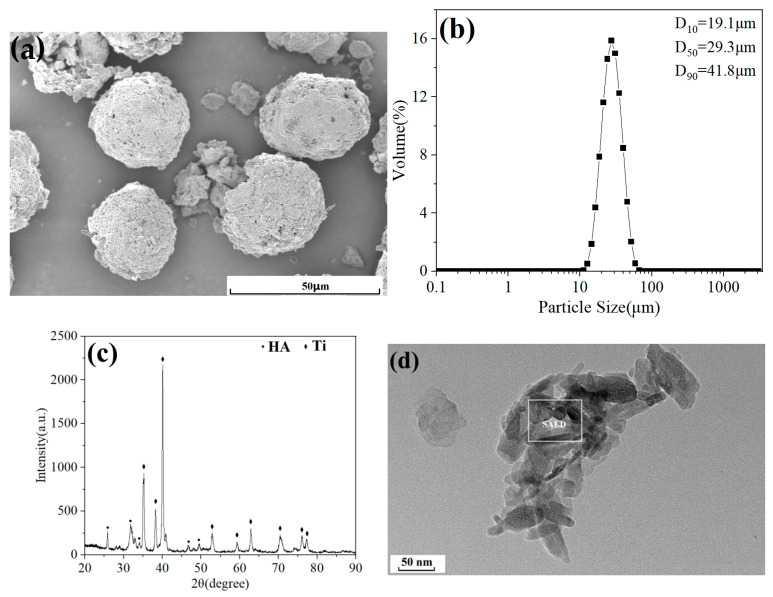

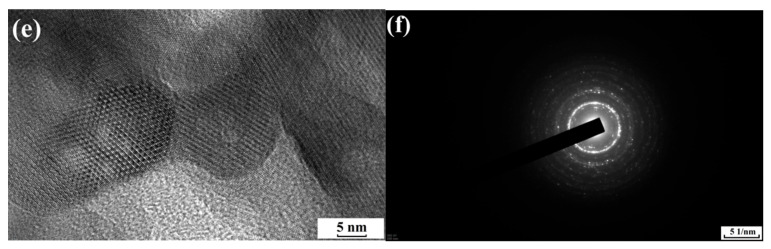
(**a**) Surface morphology; (**b**) particle size distribution; (**c**) XRD pattern; (**d**–**f**) TEM morphology of nHA/Ti composite powder.

Needle-shaped nano HA powder with an average particle size of 15 nm (Beijing Dk nano S&T Ltd., Beijing, China) and angular nano Ti powder with a mean particle size of 40 nm (Beijing Dk nano S&T Ltd., China) were selected as raw materials. Agglomeration-sintering method was used to prepare nHA/Ti composite powder with a HA: Ti mass ratio of 3:7. Figure 1 shows the surface morphology, particle size distribution, XRD pattern, and TEM morphology of nHA/Ti composite powder. The surface morphology shows that the nHA/Ti composite particles exhibits a spherical shape (Figure 1a) with a median particle size (D50) of 29.3 μm (Figure 1b). The XRD pattern (Figure 1c) confirms the exclusive presence of HA and Ti phases in the nHA/Ti composite powder, indicating no impurity formation during the preparation process. The TEM morphology of nHA/Ti composite particles (Figure 1d) reveals that the nano HA exhibits a needle-like morphology with sizes ranging between 7 and 22 nm, while the nano Ti displays an angular morphology with sizes of 30–50 nm. High-magnification TEM analysis of the nHA/Ti composite particles (Figure 1e) reveals well-defined lattice fringes, confirming the high crystallinity of both the nano HA and nano Ti starting powders. Furthermore, the absence of halo features in the corresponding selected-area electron diffraction (SAED) pattern (Figure 1f) provides conclusive evidence for the fully crystalline nature of these nanomaterials.

The deposition of individual nHA/Ti splats and coating was performed using a cold spray system, which has been described in detail elsewhere [26,30]. Ti6Al4V was used as the substrate material. Prior to individual splat deposition, the substrate surface was polished. Before coating deposition, the substrate surface was sandblasted using 700 μm alumina particles, followed by deposition of a Ti buffer layer for subsequent nHA/Ti coating deposition. The detailed deposition parameters for both individual splats and coatings are listed in Table 1.

The surface morphology of the nHA/Ti composite powder and individual splats and the surface and cross-sectional morphologies of the nHA/Ti composite coating were characterized by scanning electron microscopy (SEM, KYKY-EM8000, KYKY Technology Co., Ltd., Beijing, China). Transmission electron microscopy (Tokyo TEM, EM-2010, HR, JEOL Japan, Tokyo, Japan) was employed to analyze the microstructure and crystallographic properties of nHA/Ti composite powder. The phase composition of both the nHA/Ti composite powder and coatings was determined by X-ray diffraction (XRD, Bruker D8 Advance, Karlsruhe, Germany).

The particle size distribution of nHA/Ti composite powder was measured using a laser diffraction particle size analysis meter (GSL-1020, Liaoning Instrument Research Institute Co., Ltd., Dandong, China), and the in-flight velocity of composite particles under different temperature conditions during cold spraying was measured using a DPV eVOLUTION system (Tecnar Automation Ltd., Saint-Bruno-de-Montarville, QC, Canada).

**Figure 2:**
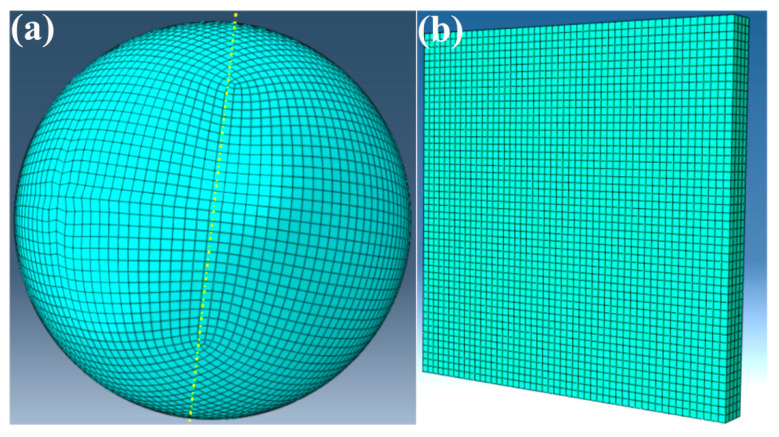
Meshing of (**a**) spherical particle; (**b**) substrate.

**Figure 3:**
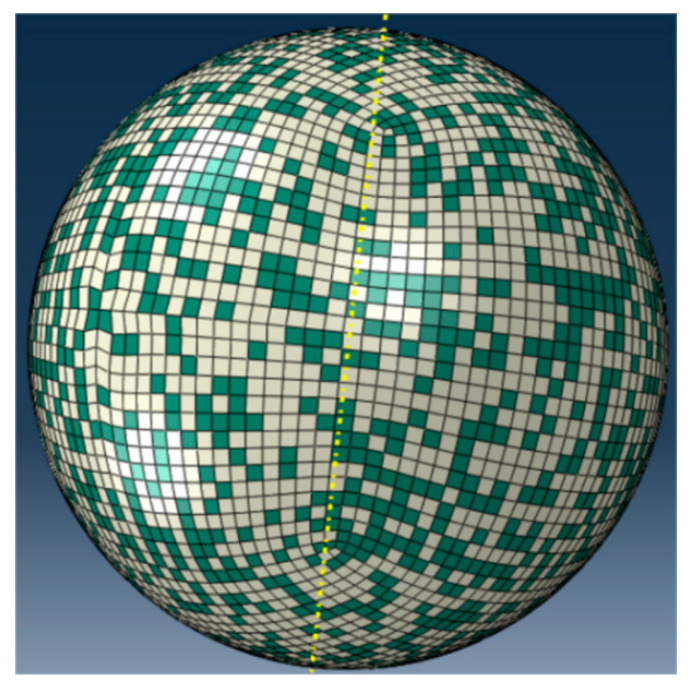
The surface morphology of a randomly distributed spherical particle.

**Figure 4:**
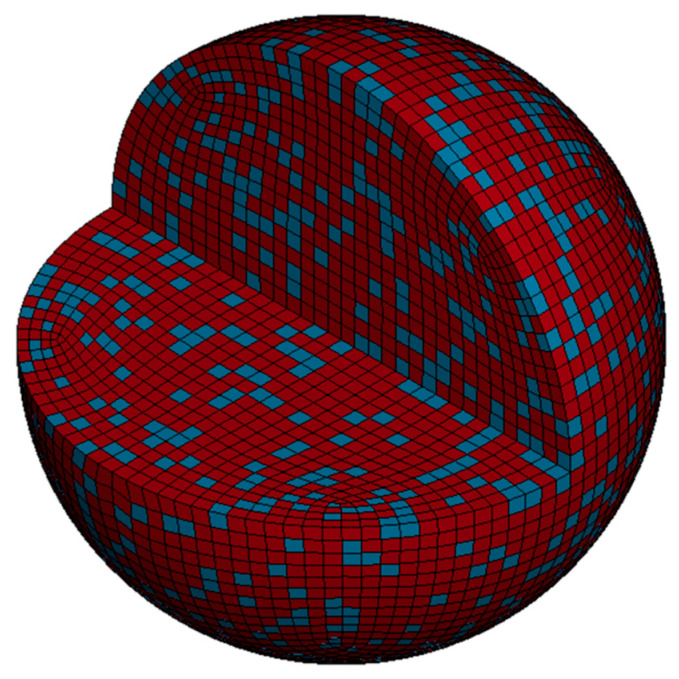
A locally sectioned view of the spherical particle model.

**Figure 5:**
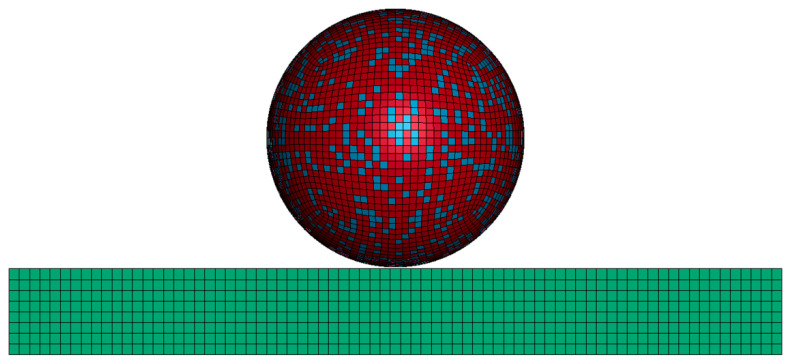
An integrated composite particle–substrate model.

In the ABAQUS/CAE module, the number of particle meshes is configured (constrained between 30,000 and 50,000) to generate a spherical particle model. The particle is then discretized using a hexahedral mesh, resulting in a fully meshed spherical particle (as shown in Figure 2a). As the substrate plate is a regular geometric model, hexahedral meshing was applied directly to the substrate, resulting in a fully meshed substrate model (as shown in Figure 2b). Through Python 3.9-based secondary development, the meshed spherical particle was randomly distributed according to a HA/Ti volume ratio of 1:1.59 (corresponding to a mass ratio of 30:70), categorizing the elements stochastically into HA and Ti phases, where yellow particles represent Ti and green particles denote HA (as shown in Figure 3). After importing the meshed composite particle model file into LS-PrePost 4.9-x64 software, a locally sectioned view of the spherical particle model could be obtained, where red particles represent Ti and blue particles denote HA (as shown in Figure 4). The established substrate model (Figure 2b) and randomly distributed spherical particle (Figure 3) were imported into LS-PrePost 4.9-x64 software to obtain an integrated composite particle–substrate model (as shown in Figure 5). For enhanced simulation accuracy, the parameters of nano-HA and nano-Ti particles in the nHA/Ti composite must be input into the LS-PrePost 4.9-x64 software, as both sets of nanoparticle parameters have been reported in the references [35].

Considering the simulation computation time, the powder feeding pressure for individual composite particle deposition simulation (with particle size set to 20 μm) was set at 2.2 MPa, with a spraying distance of 20 mm, nitrogen as the spraying gas, substrate initial temperature of 300 K, and particle initial temperature of 773 K. The deposition velocities of individual particles were set to 400 m/s, 500 m/s, 600 m/s, 700 m/s, and 800 m/s.

Particles deposited on the substrate surface are typically characterized using the particle compression ratio to quantify the degree of deformation. The particle compression ratio (η) is defined as shown in Equation (1):

η = (h_i_ − h_d_)/h_i_ (1)

where η is the particle compression ratio, h_i_ is the initial particle diameter, and h_d_ is the post-deformation height of the particle.

## 3. Results and Discussion

### 3.1. Numerical Simulation of Deposition Behavior for Individual Composite Particles at Varying Particle Velocities

**Figure 6:**
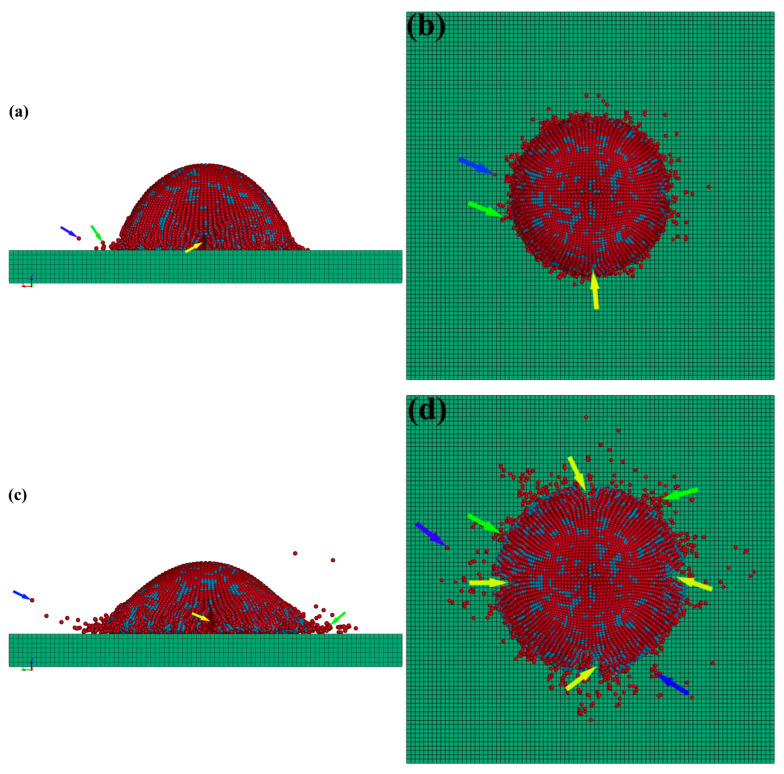

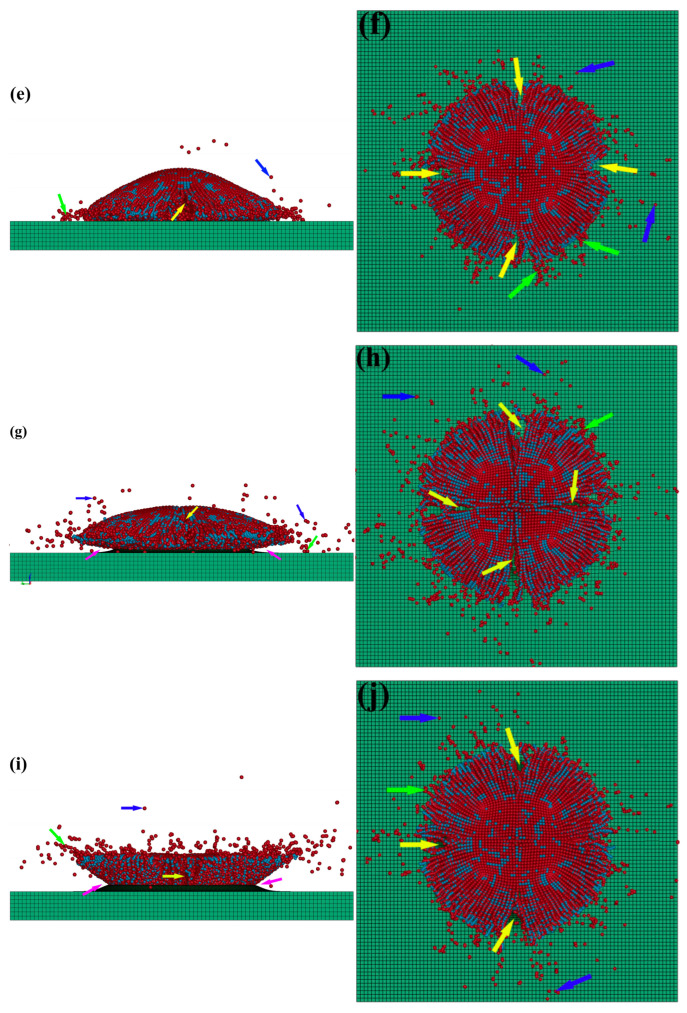
Simulated morphologies of HA-70wt.%Ti composite particles deposited under different particle velocity conditions: (**a**,**b**) 400 m/s; (**c**,**d**) 500 m/s; (**e**,**f**) 600 m/s; (**g**,**h**) 700 m/s; (**i**,**j**) 800 m/s.

Figure 6 illustrates the simulated instantaneous deformation morphologies of nHA/Ti composite particles after impacting on the substrate at velocities ranging from 400 to 800 m/s. The instantaneous simulation time was 3 × 10^−8^ s. The side views of the deformed particles are shown in Figure 6a,c,e,g,i, and the top views in Figure 6b,d,f,h,j. When the particles impacted the substrate at a relatively low velocity (400 m/s), the composite particles experienced limited deformation, largely maintaining their hemispherical morphology (Figure 6a). Only a small number of particles surrounding the deformation zone exhibited phenomena such as splattering (indicated by green arrows), rebounding-off (marked by blue arrows), or crack formation (denoted by yellow arrows). With increasing particle velocity, the deformed particles progressively evolved from hemispherical to flattened configurations (Figure 6c), while the splattering, rebounding-off and crack phenomena at the periphery of deformed particles became significantly more pronounced (Figure 6d). As the particle velocity further increased (Figure 6e,g,i), the phenomena of particle rebounding-off, splattering and cracking intensified significantly. Concurrently, the bonding between deformed particles and the substrate deteriorated progressively, leading to the formation of distinct gaps at the particle–substrate interface (indicated by purple arrows in Figure 6g,i), whose dimensions exhibited a positive correlation with impact velocity (Figure 6i). Ultimately, such interfacial failure may cause macroscopic spallation of deposited particles from the substrate surface. The compression ratios for deformed composite particles at different particle velocities (400 m/s to 800 m/s) were 37.0%, 47.0%, 59.9%, 64.3%, and 76.2%, respectively.

**Figure 7:**
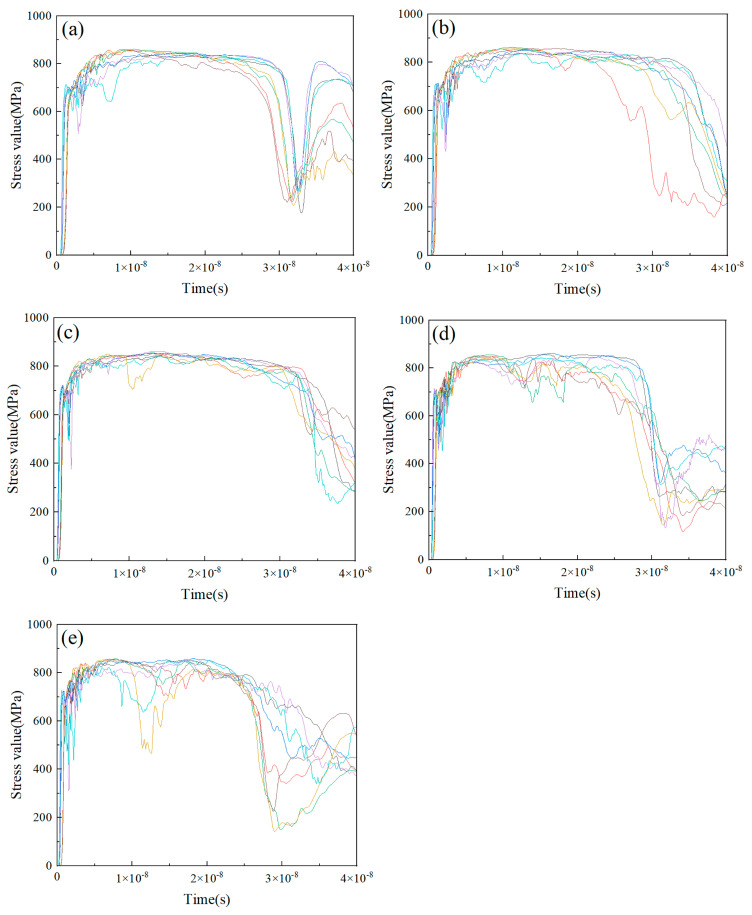
Stress curves of Ti particulate units in HA-70wt.%Ti composite particles after deposition under different particle velocity conditions: (**a**) 400 m/s; (**b**) 500 m/s; (**c**) 600 m/s; (**d**) 700 m/s; (**e**) 800 m/s.

**Figure 8:**
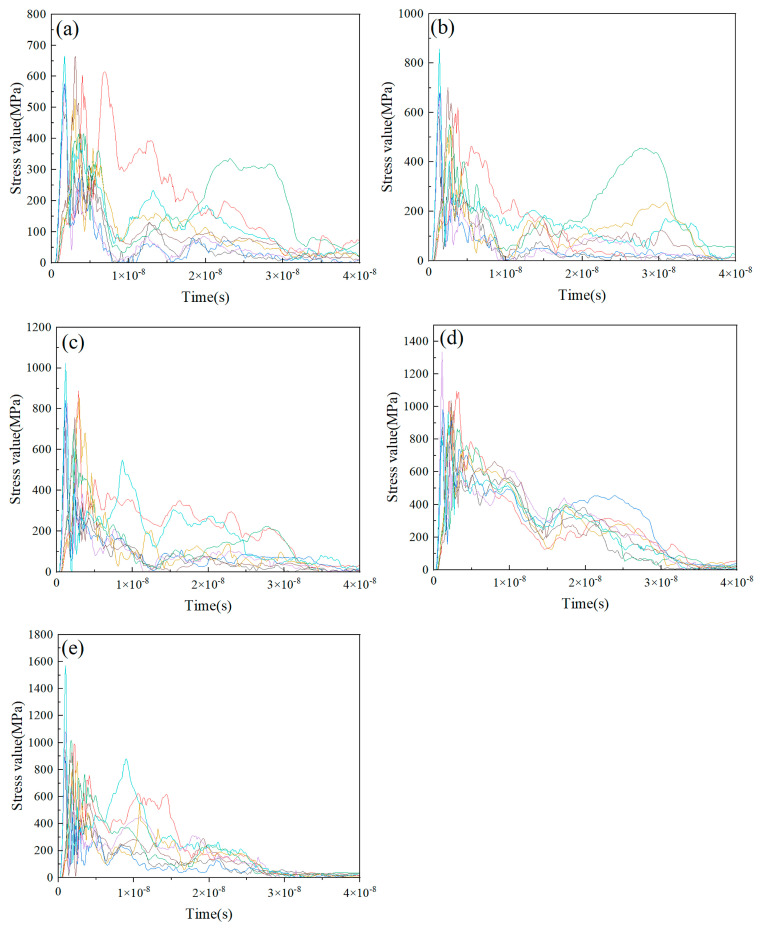
Stress curves of HA particulate units in HA-70wt.%Ti composite particles after deposition under different particle velocity conditions: (**a**) 400 m/s; (**b**) 500 m/s; (**c**) 600 m/s; (**d**) 700 m/s; (**e**) 800 m/s.

Figure 7 and Figure 8 present the stress value curves of eight randomly selected Ti and HA particles from the composite particles deposited under varying particle velocity conditions, respectively. Figure 7 revealed that the stress value of the Ti particle unit rapidly peaked during the initial collision instant, demonstrating the occurrence of adiabatic shear instability (ASI). After sustaining a stabilized stress state for a duration, the stress curve of the Ti particle unit manifested a progressive decline. This phenomenon arose from thermomechanical coupling during impact: plastic work dissipates as thermal energy, inducing thermal softening in the Ti particles and consequently leading to stress value decrease. Figure 8 showed that the stress value in the HA particle unit also rose quickly to its peak value during the initial collision phase. Meanwhile, the peak stress in the HA particle unit increased with increasing particle velocity.

### 3.2. Deposition Behavior Analysis of Cold-Sprayed Single nHA/Ti Composite Particle

**Figure 9:**
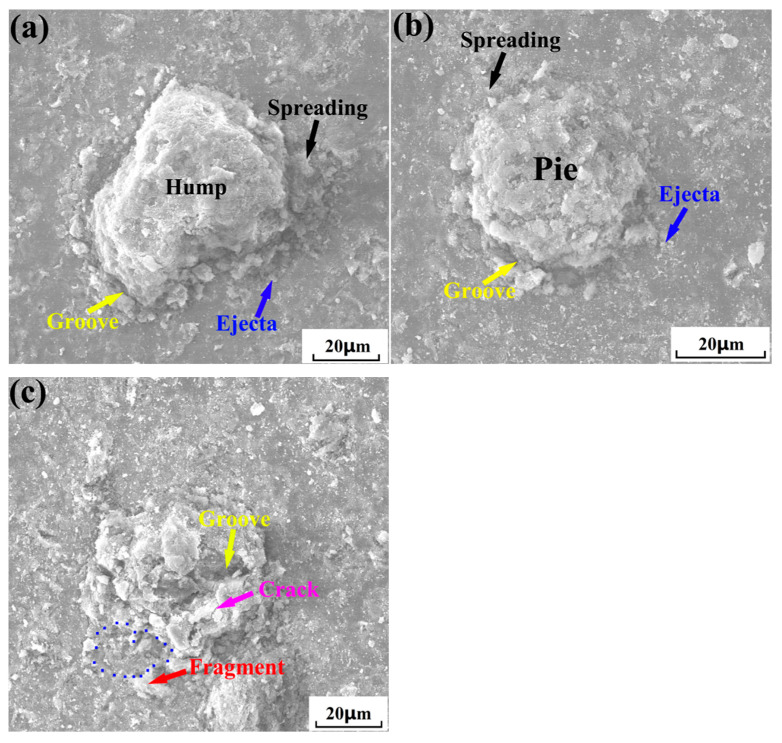
Surface morphologies of cold-sprayed single HA-70wt.%Ti composite particles at different gas temperatures: (**a**) 300 °C; (**b**) 500 °C; (**c**) 700 °C.

**Figure 10:**
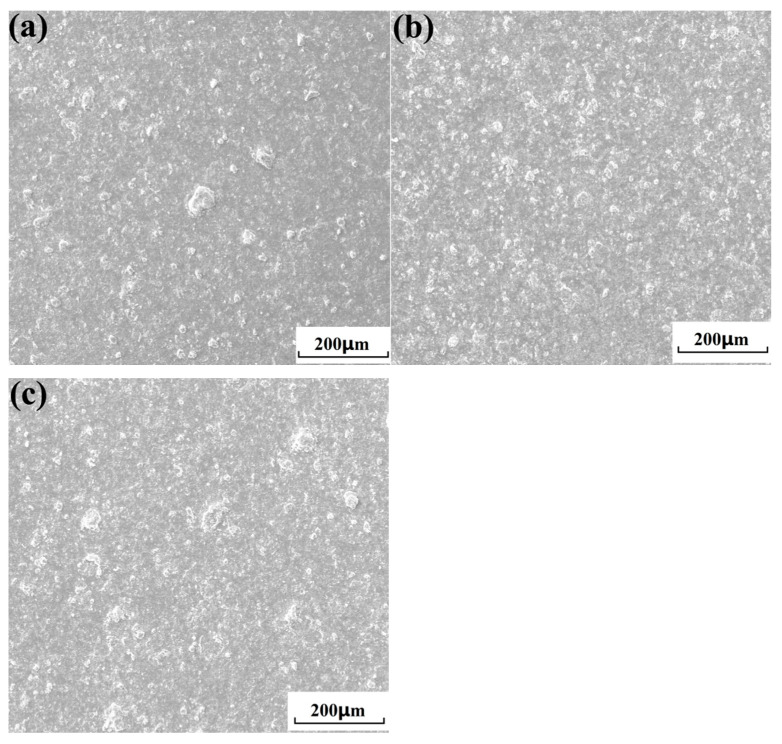
Bonding morphologies of cold-sprayed HA-70wt.%Ti composite particles at different gas temperatures: (**a**) 300 °C; (**b**) 500 °C; (**c**) 700 °C.

Figure 9 shows the surface morphologies of cold-sprayed single nHA/Ti composite particle at different temperatures. When the gas temperature was relatively low (300 °C), the composite particle after deposition deformation exhibited a hump morphology at the center (as illustrated in Figure 9a), accompanied by peripheral spreading (as indicated by the black arrow), ejecta (as indicated by the blue arrow), and groove (as indicated by the yellow arrow) phenomena around the deposited particles. Compared to the deposition characteristics of composite particles at a gas temperature of 300 °C, elevating the gas temperature to 500 °C resulted in significantly enhanced deformation and more pronounced splattering phenomena upon particle deposition on the substrate surface (Figure 9b). Furthermore, the deformed particles exhibited a greater flattening degree, accompanied by the formation of more distinct grooves around the deposition zones. Compared with the deposition behavior at gas temperatures of 300 °C and 500 °C, as the gas temperature increased to 700 °C, the particles deposited on the substrate surface exhibited more pronounced deformation (Figure 9c). Upon impact with the substrate, individual particles underwent fragmentation (as indicated by the red arrow) and cracking (as indicated by the purple arrow), accompanied by a rebound-splattering phenomenon of the fractured particles (marked with a blue dot circle). This indicated that experimental results of composite particle deposition validated simulation predictions (as shown in Figure 6). Compared to previous studies [35], individual HA/70Ti composite particles exhibited relatively smaller deformation under the same cold spraying conditions. This indicates that the variation in the ceramic phase content of the composite powder affects particle deposition deformation. The increase in the hard and brittle HA ceramic phase makes it prone to cracking and rebound/splattering during the deposition process, which in turn leads to a greater extent of deformation. Figure 10 shows the bonding morphologies of nHA/Ti composite particles deposited on the substrate at different gas temperatures. It can be observed from the Figure that as the gas temperature increased from 300 °C to 500 °C, the density of composite particles bonded to the substrate surface gradually increased. However, when the gas temperature increased to 700 °C, the particle bonding density became slightly lower than that at 500 °C. Intense particle impaction at elevated temperature caused this phenomenon, inducing severe deformation, fragmentation, and cracking, which led to partial particle rebound and splashing from the substrate surface. Meanwhile, the measured average velocities of nHA/Ti composite particles at 300 °C, 500 °C, and 700 °C were 526 m/s, 685 m/s, and 758 m/s, respectively.

### 3.3. Deposition Behavior Analysis of Cold-Sprayed Multiple nHA/Ti Composite Particles

**Figure 11:**
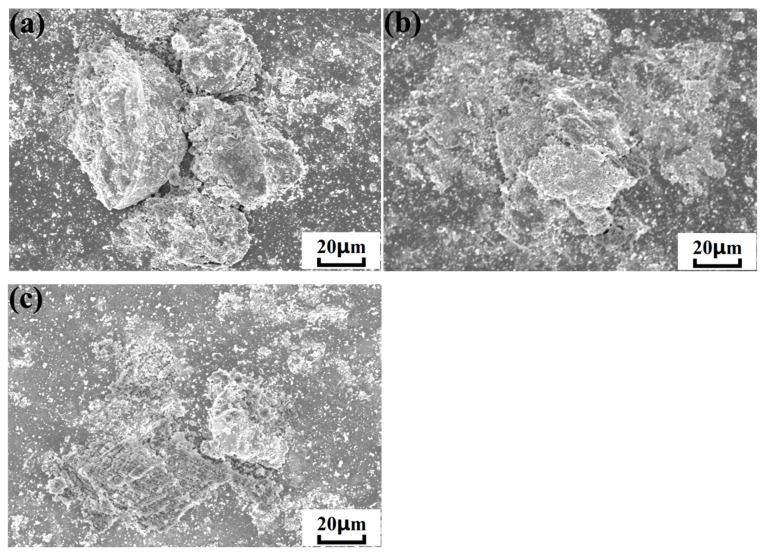
Surface morphologies of cold-sprayed multiple HA-70wt.%Ti composite particles at different gas temperatures: (**a**) 300 °C; (**b**) 500 °C; (**c**) 700 °C.

Figure 11 shows the surface morphologies of cold-sprayed multiple nHA/Ti composite particles at different temperatures. It revealed that at a relatively low gas temperature (300 °C), the deposited multiple particles exhibited limited deformation upon impact with the substrate. However, interparticle collisions resulted in noticeable splattering. As the gas temperature increased to 500 °C, the multiple particles’ deformation and flattening became more pronounced, forming near-complete stacking between particles, accompanied by moderate splattering. When the gas temperature continued to rise to 700 °C, multiple particles underwent severe deformation after deposition, and the splashing phenomenon caused by the impact of deposited particles on the substrate and the mutual collision between particles also became more serious. This was consistent with the results of particle deposition simulations, further indicating that as the gas temperature increased, the degree of particle deformation increased accordingly, as did the intensity of mutual collisions between particles. Although the degree of multi-particle deposition deformation increased with the rise in gas temperature, the bonding effect of multi-particle deposition was better at a gas temperature of 500 °C. This also indicated that the bonding effect of particle deposition did not improve monotonically with increasing gas temperature. The main reason for this is that at a gas temperature of 700 °C, the poor deformability of both HA and Ti materials led to particularly severe particle splashing during deposition, which resulted in inferior bonding quality.

### 3.4. Microstructure of Cold-Sprayed nHA/Ti Composite Coatings

**Figure 12:**
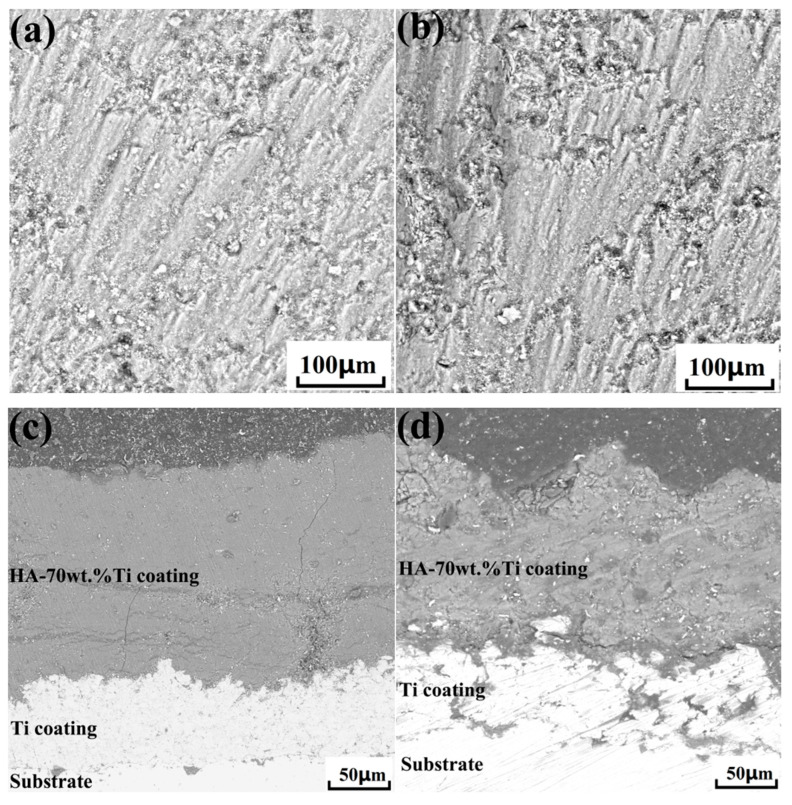
Surface and cross-sectional morphologies of cold-sprayed HA-70wt.%Ti composite coatings at different gas temperatures: (**a**,**c**) 500 °C; (**b**,**d**) 700 °C.

**Figure 13:**
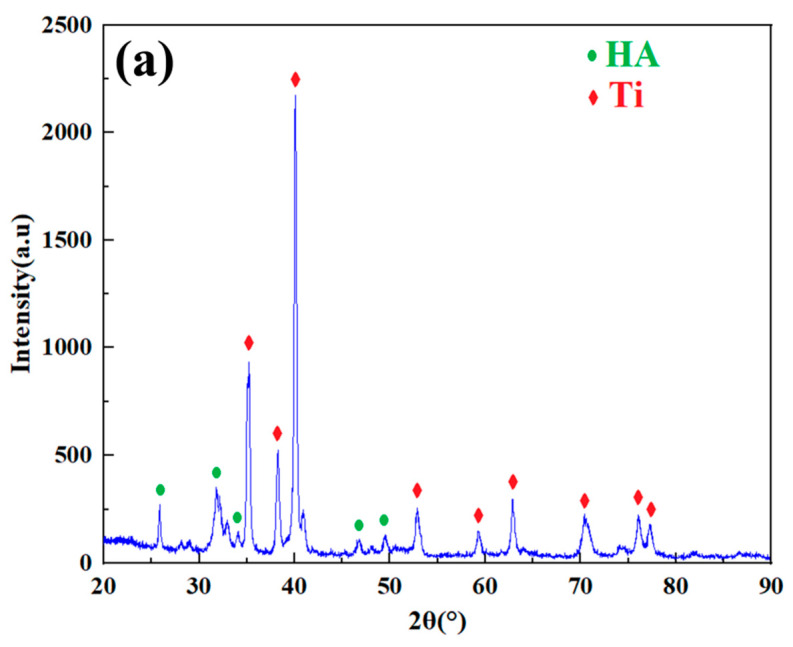

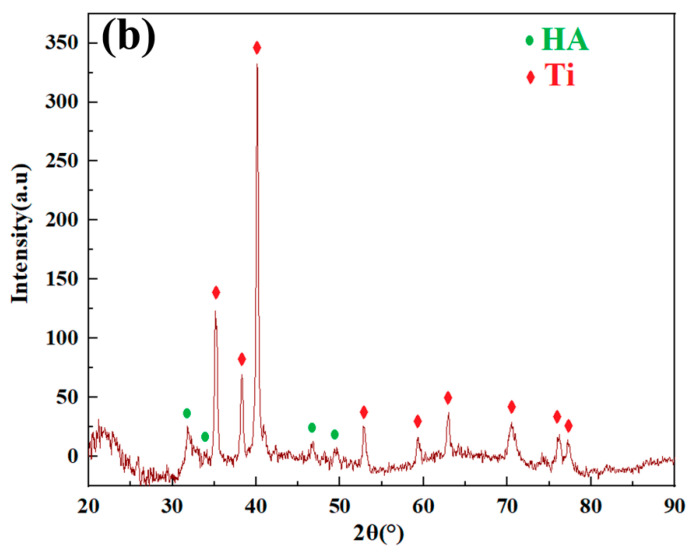
XRD patterns of HA-70wt.%Ti composite coatings at different gas temperatures: (**a**) 500 °C; (**b**) 700 °C.

Based on the analysis of particle deposition deformation and bonding, it was found that both particle deformation and bonding were poor at a gas temperature of 300 °C. Therefore, the gas temperatures selected for coating preparation were 500 °C and 700 °C. Figure 12a,b show the surface morphologies of nHA/Ti coatings at gas temperatures of 500 °C and 700 °C, respectively. It can be observed that both coatings exhibit relatively dense and rough surface structures. However, the surface of the coating fabricated at a gas temperature of 500 °C exhibited a flatter morphology than that prepared at 700 °C. Figure 12c,d show the cross-sectional morphologies of nHA/Ti coatings at gas temperatures of 500 °C and 700 °C, respectively. It can be observed that at the gas temperature of 500 °C, the coating exhibited good bonding with the Ti buffer layer, and the internal density of the coating was relatively high. However, longitudinal cracks were generated in the coating during the deposition process. When the gas temperature was 700 °C, the bonding between the coating and the Ti buffer layer was poor, and the bonding between particles inside the coating was also weak. The main reason was that at a gas temperature of 700 °C, the splashing phenomenon caused during the particle deposition process led to poor bonding between internal particles and between the coating and the substrate. The average thicknesses of nHA/Ti coatings deposited at gas temperatures of 500 °C and 700 °C were 162.43 μm and 126.11 μm, respectively. The XRD analysis patterns of nHA/Ti coatings are shown in Figure 13. It can be seen that the phases of both coatings were consistent with those of the original powder, indicating that no phase transformation or new phase formation occurred in the nHA/Ti powder particle during the deposition process.

## 4. Conclusions

In this study, the deposition behavior for cold sprayed nano-structured nHA/Ti composite coating using both experimental and numerical simulation approaches was investigated. The results of single particle deposition simulations indicated that as the particle velocity increased, the cracking phenomenon of the splat became more severe and the bond between the splat and the substrate deteriorated. As the gas temperature increased, the degree of particle deformation and the particle impact velocity both increased. The results from cold-sprayed multi-particle deposition experiments indicated that as the gas temperature increased, the extent of interparticle impact and deformation correspondingly increased. The coating fabricated at a gas temperature of 500 °C exhibited a flatter surface morphology and better bonding with the Ti buffer layer than that prepared at 700 °C. The phases present in the coatings were consistent with those of the original powder.

## Figures and Tables

**Table 1:** The detailed deposition parameters for both individual splats and coatings.

Parameters	Unit	Value
Accelerating nitrogen gas pressure	MPa	2.0
Powder-feeding nitrogen gas pressure	MPa	2.2
Gas temperature for individual nHA/Ti splats	°C	300 ± 10, 500 ± 10, 700 ± 10
Gas temperature for nHA/Ti coating	°C	500 ± 30, 700 ± 30
Gas temperature for Ti buffer layer	°C	200 ± 30
Transverse speed for individual nHA/Ti splats	mm/s	500
Transverse speed for nHA/Ti coating	mm/s	30
Transverse speed for Ti buffer layer	mm/s	150
Spray distance	mm	20

## Data Availability

The data that support the findings of this study are available from the corresponding author upon reasonable request.
